# Adolescent dieting behaviour: associations with physical activity and screen-time behaviours

**DOI:** 10.1186/2050-2974-1-S1-O66

**Published:** 2013-11-14

**Authors:** Amanda Dando, Peter Kremer, John W Toumbourou, Joanne Williams, George Patton

**Affiliations:** 1 School of Psychology, Deakin University, Australia; 2 School of Exercise and Nutrition Sciences, Deakin University, Australia; 3 School of Psychology and Centre for Mental Health and Wellbeing Research, Deakin University, Australia; 4 Murdoch Children's Research Institute, Australia; 5 Centre for Adolescent Health, Royal Children's Hospital, Australia; 6 Department of Paediatrics, University of Melbourne, Australia

## 

Adolescent dieting remains a relatively neglected area despite evidence of its relationship with significant public health concerns such as obesity, eating disorders and psychiatric problems. This cross-sectional study examines associations between physical activity (PA) and screen-time behaviours (STB) as predictors of dieting behaviour among 7648 Australian adolescents aged 10-14 years, using data from the Healthy Neighbourhoods Study. Participants completed an online survey and answered questions examining dieting behaviour using the Adolescent Dieting Scale, PA, STB, weight and pubertal status, family risk factors and peer victimisation. Based on published cut-offs, 69.7% of adolescents were 'minimal/non-dieters' and 30.3% were 'moderate/extreme dieters'. Logistic regression analysis found that being female and meeting national PA and STB guidelines were significant correlates of adolescent dieting. These associations persisted with the inclusion of biological and social predictors into the model, further revealing high levels of pubertal development, poor family management, high family conflict, parental over-control and peer victimisation were associated with increased the odds for adolescent dieting. Interaction analyses revealed that older females were 50% more likely to be dieters than their younger counterparts and older males. Findings indicate that potentially unhealthy dieting practices in early adolescence are associated with meeting national PA and STB guidelines.

This abstract was presented in the **Understanding and Treating Eating Pathology** stream of the 2013 ANZAED Conference.

